# Dataset of copper pipes corrosion after exposure to chlorine

**DOI:** 10.1016/j.dib.2018.05.023

**Published:** 2018-05-19

**Authors:** Fernando García-Ávila, Gabriel Bonifaz-Barba, Silvana Donoso-Moscoso, Lisveth Flores del Pino, Lía Ramos-Fernández

**Affiliations:** a Universidad Nacional Agraria La Molina, Lima, Perú; b Facultad de Ciencias Químicas, Universidad de Cuenca, Ecuador; c Departamento de Biociencias, Grupo Nutrición Alimentación y Salud, Universidad de Cuenca, Ecuador; d Centro de Investigación en Química, Toxicología y Biotecnología Ambiental del Departamento Académico de Química de la Facultad de Ciencias de la UNALM, Lima, Perú; e Departamento Académico de Recursos Hídricos, UNALM, Lima, Perú

## Abstract

This article presents data on corrosion and dissolved copper in copper tubes that transport drinking water in domiciles of the Azogues city, Ecuador. Corrosion tests were performed using copper coupons exposed to water with different concentrations of free chlorine for 30, 60, 90 and 180 days. The determination of the copper corrosion rate exposed in chlorine was carried out by means of gravimetric tests. With weight loss data, the corrosion rate was determined. By means of static immersion tests, copper release of coupon surface was determined. In the obtained data it was observed that the corrosion rate and the release of copper increases with the chlorine concentration. This data is beneficial for drinking water companies and building builders by providing information on the corrosion and leaching behavior of copper pipes when exposed to chlorine and is useful for predicting the service life copper pipes. In addition, it could allow assessing the health risk by consuming water with copper in solution.

**Specifications Table**

**Table:** 

Subject area	Environmental Science
More specific subject area	Corrosion of metals, Drinking water chemistry
Type of data	Table and figure
How data was acquired	The corrosion rate by weight difference of the copper coupons in dynamic and static immersion tests after a period of exposure was obtained. The weight measurements were carried out on a Sartorius analytical balance. The copper release with the HACH DR/2500 Spectrophotometer was measured. Free chlorine was measured with HACH DR/890 Colorimeter.
Data format	Raw, analyzed
Experimental factors	Concentration of free chlorine, exposure time.
Experimental features	The experimental tests were carried out in three places: a) Drinking water treatment plant (dynamic immersion), in a channel after the filtration without chlorine and a channel after chlorination; b) Domicile, a corrosion test rack (dynamic immersion) was installed; c) Laboratory, beakers with solutions of calcium hypochlorite (static immersion). General corrosion tests according to ASTM standard G1–03 were conducted.
Data source location	Azogues, Ecuador 2°44'22" S, 78°50'54" O
Data accessibility	Data are available in the article.

**Value of the data**

• Corrosion potential of the copper pipes that transport drinking water were related to the exposure to chlorine.
• Corrosion rate can be used to predict the useful life of copper pipes until the failure of the components in service and is of interest to drinking water companies.
• The data will be useful to assess the health risk due to consumption of drinking water with dissolved copper.
• The data can be used by other researchers to develop models for the release of copper by the effect of free chlorine in copper pipes that transport drinking water.

## 1 Data

The data presented in this article deals with the copper pipes corrosion due to the effect of free chlorine present in drinking water distributed in the Azogues city, Ecuador. Corrosion is a major problem due to the destruction of various materials, especially metals [1]. It is necessary to ensure a high degree of chemical compatibility between pipe construction materials and operating fluids, such as potable water to prevent corrosion of the material [2] and health risk of drinking water consumption related to heavy metals, in this case due to copper [3], [4]. The materials of pipes, fittings and valves in distribution networks deteriorate due to corrosive water and cause some health, aesthetic and economic problems [5].

These data were determined, once the corrosion indexes were calculated in the drinking water distribution network of the Azogues city, Ecuador [6]. Formulas for determining these corrosion rates do not include residual chlorine. Authors such as [7], [8], [9], [10] mention that chlorine influences the corrosion of copper pipes that carry potable water. Reason for which the data of this investigation allow to verify the influence of the free chlorine in the copper pipes corrosion.

The data included in this document indicate the copper corrosion rate and release rate of copper pipes that carry drinking water.

**Fig. 1:**
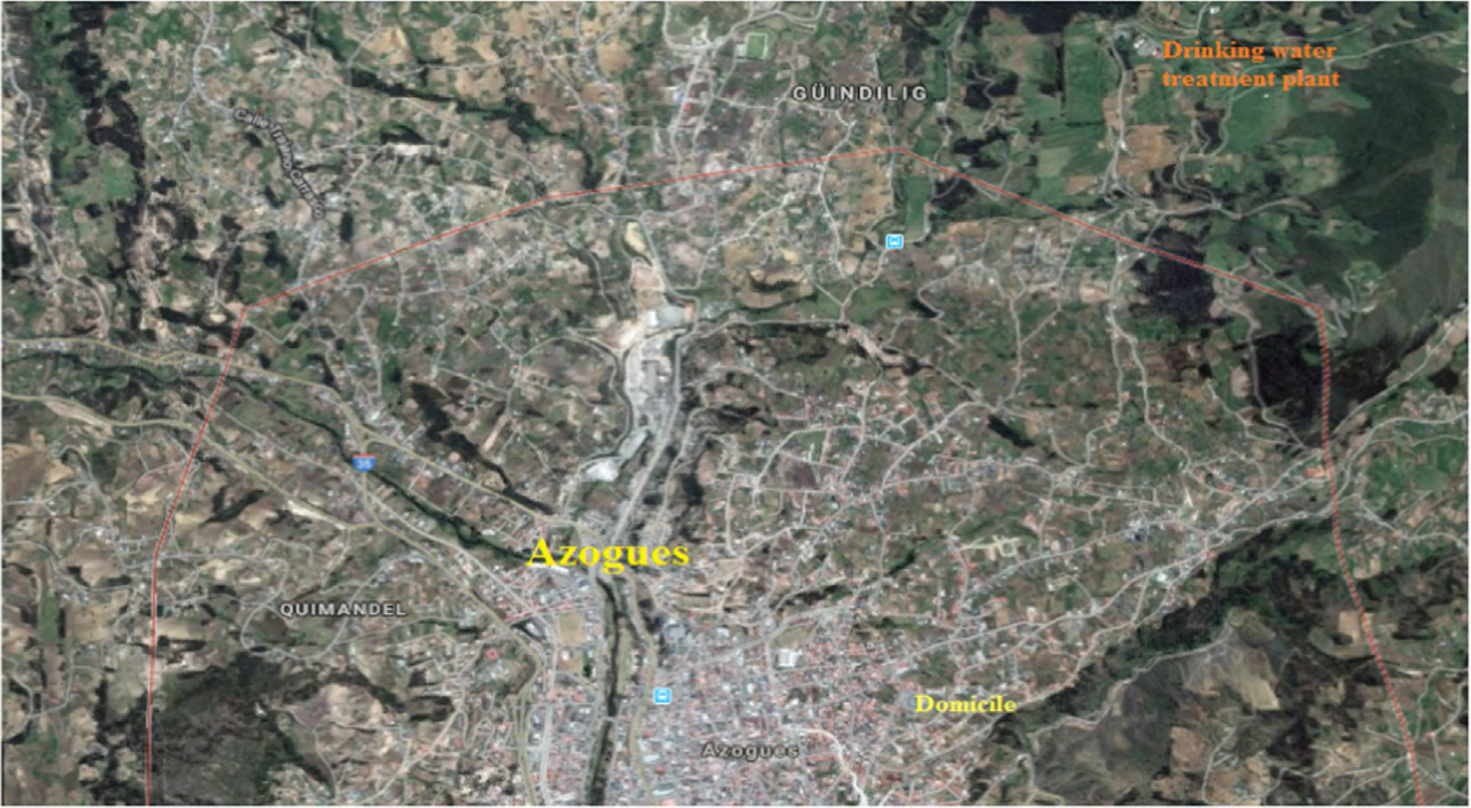
Map and location of Azogues city.

Azogues city where the study was realized is shown in Fig. 1.

The data shared in this article is presented below:

### 1.1 Weight loss in coupons

The measurements were carried out on coupons installed in the drinking water treatment plant (DWTP). The coupons were installed in a channel after the filtration that does not have chlorine, after an exposure period, the weight loss was determined for each time (Table 1).

**Table 1:** Weight loss of coupons for each exposure time submerged in water without chlorine (0 mg/L Cl_2_) in the DWTP.

**No.**	**Initial weight (mg)**	**Final weight (mg)**	**Weightloss (mg)**	**Time (days)**	**Area (cm^2^)**	**Loss of weight by area and time (mg/days.cm^2^)**
1	9057.20	9050.10	7.1	30	21.15	0.01119
2	10045.90	10039.40	6.5	30	21.15	0.01024
3	9867.40	9853.60	13.8	60	21.15	0.01087
4	9084.20	9072.30	11.9	60	21.15	0.00938
5	9994.30	9974.90	19.4	90	21.15	0.01019
6	9172.10	9155.00	17.1	90	21.15	0.00898
7	9208.00	9175.10	32.9	180	21.15	0.00864
8	9782.40	9748.10	34.3	180	21.15	0.00901

Coupons were also installed in a channel after chlorination in the treatment plant. After a period of exposure, the weight loss was determined for each time (Table 2).

**Table 2:** Weight loss of coupons for each exposure time submerged in chlorinated water (0.85 mg/L Cl_2_) in the DWTP.

**No.**	**Initial weight (mg)**	**Final weight (mg)**	**Weightloss (mg)**	**Time (days)**	**Area (cm^2^)**	**Loss of weight by area and time (mg/days.cm^2^)**
1	9194.80	9161.30	33.50	30	21.15	0.05280
2	9867.81	9832.00	35.81	30	21.15	0.05644
3	9670.20	9620.00	50.20	60	21.15	0.03956
4	9201.20	9151.20	50.00	60	21.15	0.03940
5	10038.79	9963.70	75.09	90	21.15	0.03945
6	9538.10	9466.60	71.50	90	21.15	0.03756
7	9112.70	9035.60	77.10	180	21.15	0.02025
8	9765.80	9674.30	91.50	180	21.15	0.02403

At a domicile, coupons were installed in a corrosion test rack that was connected to the household's drinking water network. After of 30, 60, 90 and 180 days the weight loss was determined (Table 3).

**Table 3:** Weight loss of the coupons for each exposure time submerged in chlorinated water (0.37 mg/L Cl_2_) in a domicile.

**No.**	**Time (days)**		**Initial weight (mg)**	**Final weight (mg)**	**Weight loss (mg)**	**Area (cm^2^)**	**Loss of weight by area and time (mg/days.cm^2^)**
1	30	9230.00		9218.80	11.2	21.15	0.01765
2	30	9999.10		9986.30	12.8	21.15	0.02017
3	60	9225.40		9208.70	16.7	21.15	0.01316
4	60	9275.50		9258.40	17.1	21.15	0.01348
5	90	9692.00		9658.80	33.2	21.15	0.01744
6	90	9601.00		9570.00	31	21.15	0.01629
7	180	9247.50		9206.30	41.2	21.15	0.01082
8	180	8923.50		8884.80	38.7	21.15	0.01017

### 1.2 Corrosion rate

The corrosion rate (Table 4) was calculated in milliliters per year (mpy) using Eq. (1) in accordance with ASTM G1-03 [11], [12].

(1) $$\mathrm{CR}=\frac{\mathrm{K W}}{\mathrm{A T D}}$$

Where: CR is Corrosion Rate (mpy), K is the corrosion rate constant (3.45 × 10^6^), W is the coupon weight loss (g), A is the coupon area (cm^2^), t is the exposure time (h), D is the copper density (8.94 g/m^3^) [11]. The weight loss of Table 1, Table 2, Table 3 was used in Eq. (1).

**Table 4:** Corrosion rate in mpy for different concentrations of free chlorine.

**No.**	**Time (days)**	**Corrosion rate en mpy**		
		**Before Chlorination(0 mg/L Cl**_**2**_**)**	**After Chlorination (0.37 mg/L Cl**_**2**_**)**	**Corrosion test Rack (0.85 mg/L Cl**_**2**_**)**
1	30	0.180	0.849	0.284
2	30	0.165	0.907	0.324
3	60	0.175	0.636	0.212
4	60	0.151	0.634	0.217
5	90	0.164	0.634	0.280
6	90	0.144	0.604	0.262
7	180	0.139	0.326	0.174
8	180	0.386	0.163	0.163

### 1.3 Copper release in coupons

In the laboratory, coupons were installed in beakers with solutions of different concentration of calcium hypochlorite. The solution of hypoclorite every 2 days for 0.25 and 0.5 mg/L Cl_2_ was changed; every 3 days for 0.75 and 1.0 mg/L Cl_2_ and every 4 days for 2.0 and 5.0 mg/L Cl_2_; which is the average time that remains chlorine in a pipe with stagnant water [10], [13]. In each change the copper release was measured (Table 5).

**Table 5:** Measurement of copper released at different concentrations of chlorine at different time intervals.

	**Copper release (µg/L)**							
**No. day**	**0.25 mg/L Cl**_**2**_	**0.50 mg/L Cl**_**2**_	**No. day**	**0.75 mg/L Cl**_**2**_	**1.0 mg/L Cl**_**2**_	**No. day**	**2.0 mg/L Cl**_**2**_	**5.0 mg/L Cl**_**2**_
2	149.1	177.6	3	121.9	114.8	2	191.2	286.2
4	214.9	138.6	6	186.9	229.2	6	348.4	295.0
6	178.0	154.8	9	122.2	173.6	10	435.9	259.9
8	160.4	153.9	12	153.3	155.3	14	238.6	302.0
10	121.5	190.3	15	143.1	206.5	18	263.6	305.0
12	145.1	173.3	18	145.4	261.6	22	219.3	246.1
14	109.2	128.6	21	142.8	123.5	26	166.6	271.5
16	132.0	152.4	24	131.0	186.1	30	252.5	295.3
18	123.8	159.6	27	128.6	152.5			
20	133.3	116.7	30	157.2	176.2			
22	171.4	123.8						
24	133.3	76.2						
26	157.2	121.3						
28	158.2	135.8						
30	137.4	142.2						

**Fig. 2:**
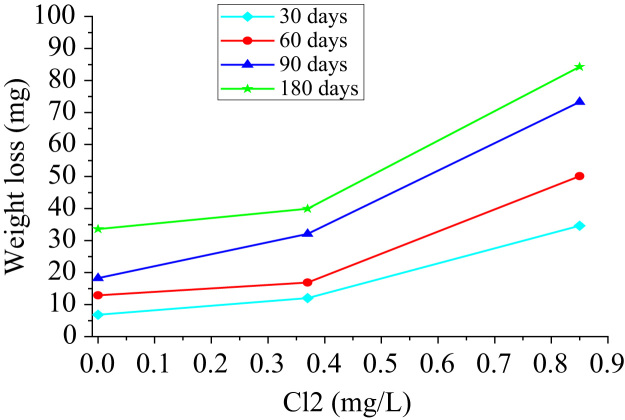
Relationship of the weight loss with the concentration of chlorine.

With those data obtained from the weight loss of each coupon, a dispersion graph was made in which the trend of weight loss was observed as a function of the chlorine concentration for each exposure period. Weight variation presents a proportional relation with the chlorine concentration for the different exposure times (Fig. 2). While higher the concentration of chlorine in the water, the copper coupon tends to lose a large part of its surface generating a uniform variation in the coupons final weight.

**Fig. 3:**
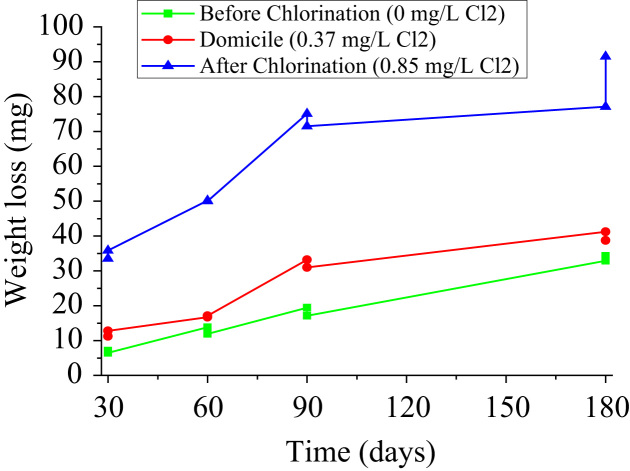
Relationship of the weight loss with the exposure time.

The trend of weight loss was observed as a function of exposure time for different free chlorine concentrations (Fig. 3). The greater the exposure time of the coupon in water with certain mg/L Cl_2_, the copper coupon tends to lose a greater part of its surface.

**Fig. 4:**
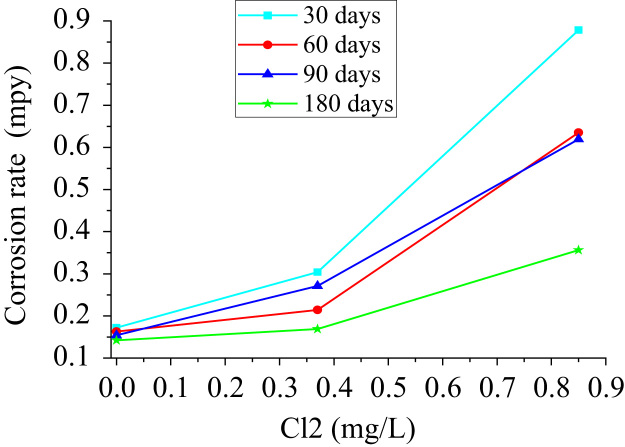
Relationship of the corrosion rate with the concentration of chlorine.

When making a dispersion diagram, the proportional tendency of the corrosion rate was observed as a function of the chlorine concentration for each period of exposure (Fig. 4). Therefore, the higher the concentration of chlorine in drinking water, the copper pipes have a tendency to increase the rate of corrosion.

**Fig. 5:**
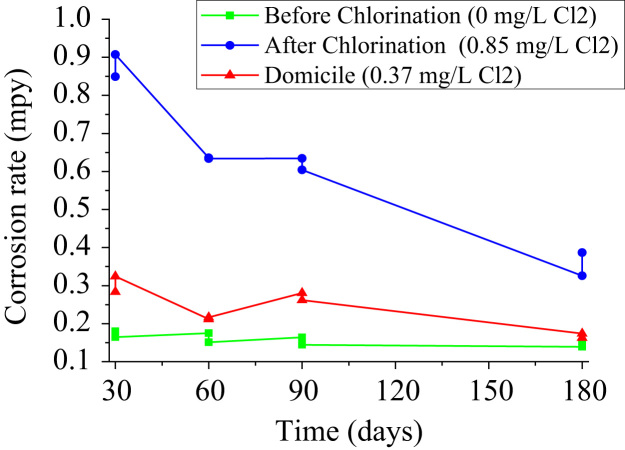
Relationship of the corrosion rate with the exposure time.

The corrosion rate tends to decrease depending on the time of exposure (Fig. 5), due to the passivation that occurs on its surface that decreases corrosion [14].

**Fig. 6:**
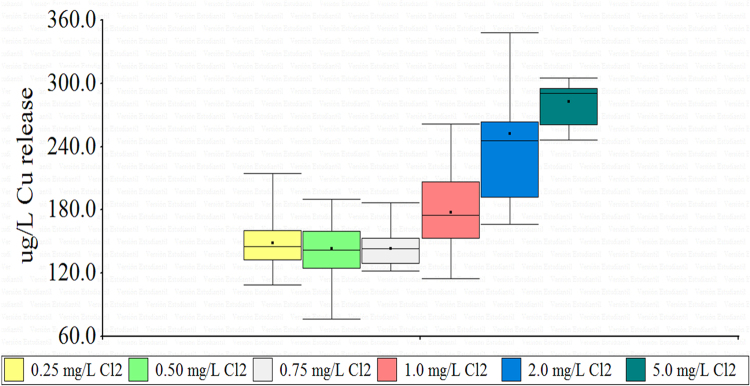
Release of copper in drinking water at different concentrations of free chlorine.

Chlorine concentrations between 0.25 to 1 mg/L in water produce a slight variation in dissolved copper concentration. By increasing the chlorine concentration to 2 and 5 mg/L, a greater copper release is generated (Fig. 6).

## 2 Experimental design, materials and methods

### 2.1 Study area description

The Azogues city is located south of the Republic of Ecuador, its geographic coordinates are: latitude 2° 44'22 "S, longitude: 78° 50'54" W, they cover an area of ​​approximately 1200 km^2^, the average altitude of the city is 2518 m above sea level, the average temperature is 17 ° C. Fig. 1 shows the location of the drinking water treatment plant.

### 2.2 Experimental design

**Fig. 7:**
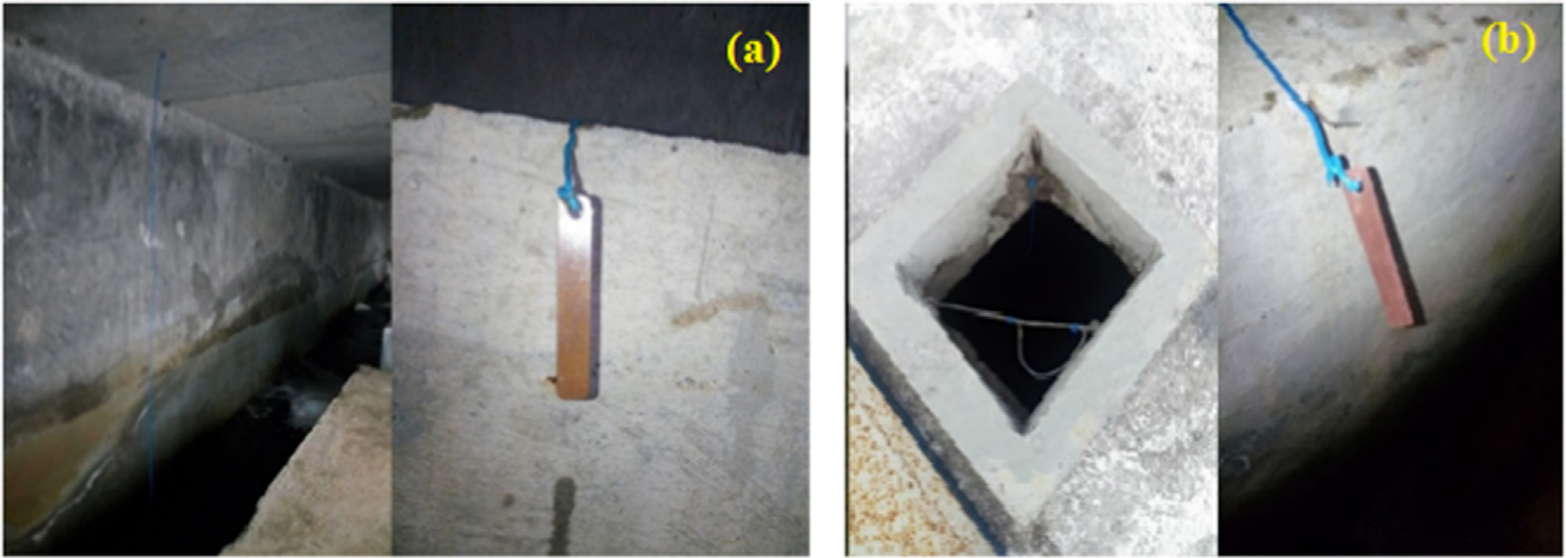
Installation of copper coupons in the DWTP. (a) Before chlorination (b) After chlorination.

In the treatment plant, the coupons were immersed in a channel after filtration with 0 mg/L Cl_2_ (Fig. 7a). Others coupons were also immersed in a tank after chlorination with an average concentration of 0.85 mg/L Cl_2_ (Fig. 7b). The coupons were suspended with nylon thread to minimize changes in coupon composition and were left for 30, 60, 90 and 180 days.

**Fig. 8:**
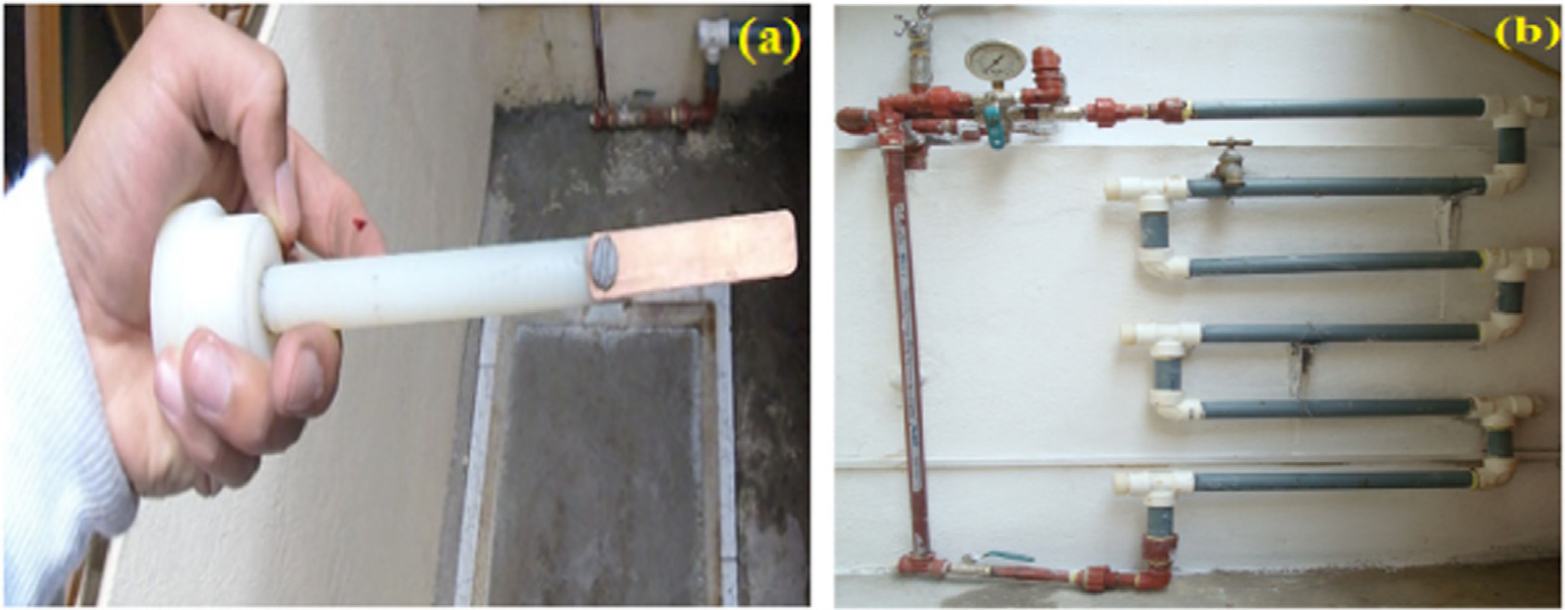
Installation of copper coupons in a domicile (a) Coupon holder (b) Corrosion test rack.

The coupons were fastened in a coupon holder (Fig. 8a), finally the holder was installed in the corrosion test rack (Fig. 8b). The coupon was left exposed to water for 30, 60, 90 and 180 days. After each trial period, were removed the coupons from the corrosion test rack and subsequently cleaned according to ASTM G1-03 [11]. Finally, the coupons were weighed to determine the weight loss and determine the corrosion rate according to Eq. 1.

**Fig. 9:**
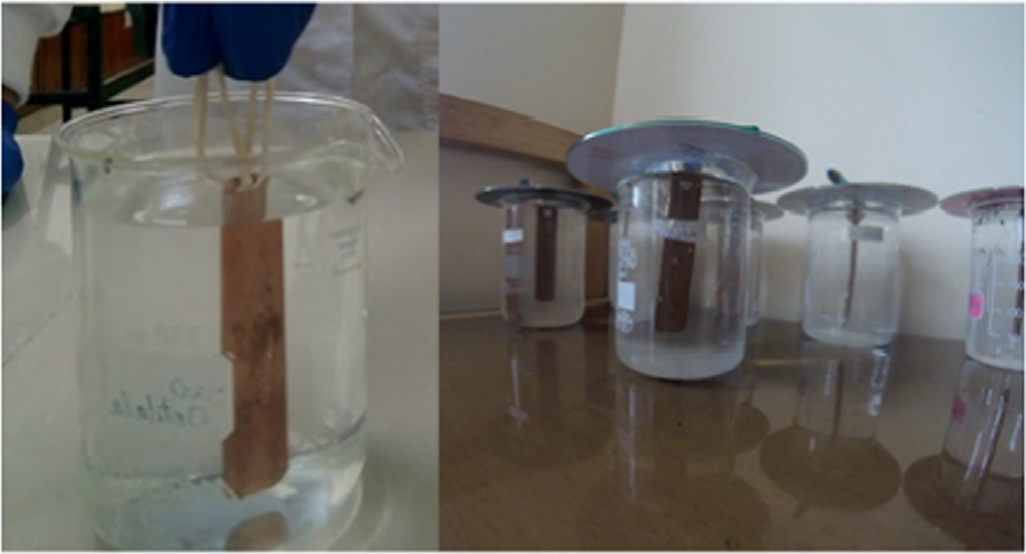
Static immersion test Coupons submerged in calcium hypochlorite solution.

For the static immersion test, potable water with the chlorine concentrations prepared above was placed in 250 ml beakers, as shown in the Fig. 9. A pre-weighed copper coupon was placed in each beaker. The solutions were changed through the emptying and filling protocol in different time intervals. Solutions with concentrations of 0.25 mg/L and 0.5 mg/L of Cl_2_ were changed every 48 h; concentrations of 0.75 and 1.0 mg/L Cl_2_ every 72 h and high concentrations solutions 2.0 and 5.0 mg/L Cl_2_ every 96 h; during a 30-day exposure time. The water resting times were used to evaluate the common scenarios of a pipe system [10], [11], [12], [13]. In this way it was intended to maintain a chlorine concentration in each beaker.

### 2.3 Materials and methods

Coupons were made from rigid K-type copper pipes used by the municipal company of the Azogues city in the residential connections. Copper coupons were prepared out in accordance with the requirements of ASTM G1-03 [11], [15]. The coupon holder was constructed of grilon with a diameter of 1.27 cm and a length of 7.62 cm (Fig. 8a). The corrosion test rack was constructed of materials that do not contribute to corrosion or cause an inhibition of corrosion. Therefore, 2.54 cm diameter PVC tubes were used. The proposed design presented six horizontal sections of 70 cm and six vertical sections of 20 cm. The right angles of the system were designed to insert the coupon holders (Fig. 8b).

Weight measurements were carried out in a Sartorius analytical balance. In this way it was possible to analyze small changes in the mass difference before and after the coupons were installed in each test site.
